# Deterministic Versus Nondeterministic Optimization Algorithms for the Restricted Boltzmann Machine

**DOI:** 10.47852/bonviewjcce42022789

**Published:** 2024-05-23

**Authors:** Gengsheng L. Zeng

**Affiliations:** 1 Department of Computer Science, Utah Valley University, USA

**Keywords:** restricted Boltzmann machine, Hopfield network, deterministic optimization, nondeterministic optimization

## Abstract

A restricted Boltzmann machine is a fully connected shallow neural network. It can be used to solve many challenging optimization problems. The Boltzmann machines are usually considered probability models. Probability models normally use nondeterministic algorithms to solve their parameters. The Hopfield network which is also known as the Ising model is a special case of a Boltzmann machine, in the sense that the hidden layer is the same as the visible layer. The weights and biases from the visible layer to the hidden layer are the same as the weights and biases from the hidden layer to the visible layer. When the Hopfield network is considered a probabilistic model, everything is treated as stochastic (i.e., random) and nondeterministic. An optimization problem in the Hopfield network is considered searching for the samples that have higher probabilities according to a probability density function. This paper proposes a method to consider the Hopfield network as a deterministic model, in which nothing is random, and no stochastic distribution is used. An optimization problem associated with the Hopfield network thus has a deterministic objective function (also known as loss function or cost function) that is the energy function itself. The purpose of the objective function is to assist the Hopfield network to reach a state that has a lower energy. This study suggests that deterministic optimization algorithms can be used for the associated optimization problems. The deterministic algorithm has the same mathematical form for the calculation of a perceptron that consists of a dot product, a bias, and a nonlinear activation function. This paper uses some examples of searching for stable states to demonstrate that the deterministic optimization method may have a faster convergence rate and smaller errors.

## 1. Introduction

**Figure 1:**
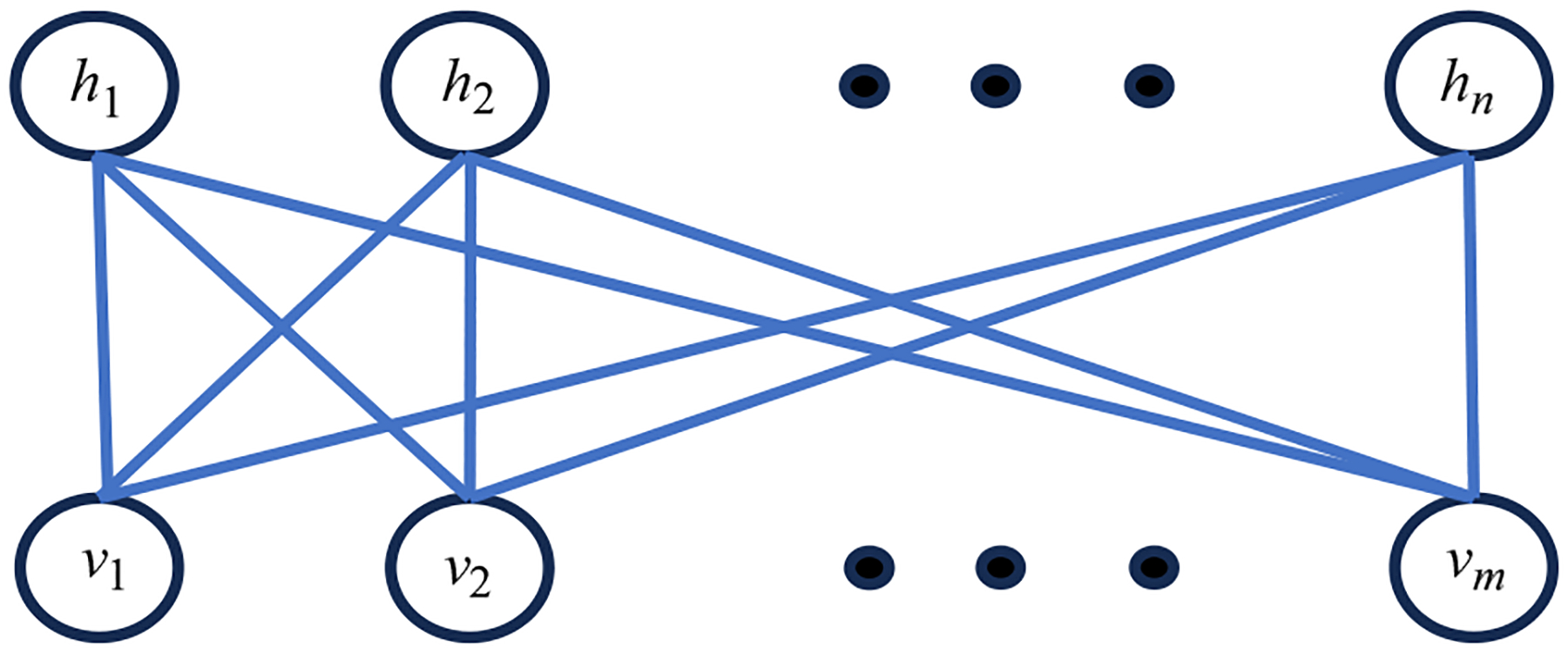
A restricted Boltzmann machine (RBM) consists of two layers: the visible layer v and the hidden layer h

A restricted Boltzmann machine (RBM) is a fully connected neural network consisting of two layers: the visible layer and hidden layer, respectively, as shown in Figure 1 [1–8]. The neurons in the visible layer are labeled as $v_{1},v_{2},\ldots ,v_{m}$, and the neurons in the hidden layer are labeled as $h_{1},h_{2},\ldots ,h_{n}$. All neurons are fully connected between the two layers. However, the neurons are not connected within each layer, which is an additional requirement dictated by the word “restricted.” In unrestricted Boltzmann machines, on the other hand, we allow connections between the neuron connections in the hidden layer [9–11].

A common RBM is binary valued. In other words, $v_{1},v_{2},\ldots ,v_{m},h_{1},h_{2},\ldots ,h_{n}\in \left\{0,1\right\}$ or $v_{1},v_{2},\ldots ,v_{m},h_{1},h_{2},\ldots ,\;h_{n}\in \left\{-1,1\right\}$. An RBM is associated with an energy function, which is a quadratic form. To use an RBM, a problem is formulated as an energy function (also known as, an objective function, a cost function, or a loss function). This energy function is formed according to the task to be performed [12–17].

A unique feature of the RBMs is their parallel implementations by, for example, using the Field Programmable Gate Array (FPGA) technology [18–23]. Therefore, they can be used to solve the NP-hard problems once the NP-hard problems are represented by well-defined energy functions. Many NP-hard problems can be formulated in a combinatorial nature.

In most machine learning tasks, input data and desired output data (known as targets or labels) are given to train the neural network parameters (known as weights and biases), according to a user-specified cost function, which defines the distance between the desired target and the model produced result. However, in a typical RBM application, we do not use the input data and the desired output data to train the network parameters. The network parameters are calculated through a design procedure. If there is no training, there is no need for an objective function.

The energy function in the context of Boltzmann machines is inherent to the network weights and biases; the weights connect the visible layer and the hidden layer. The energy function can be used as an objective function. However, this objective function is not quite the same as the objective function that is used commonly in machine learning. The common objective function in machine learning is used for network training to find the weights and biases. On the other hand, the energy function of a Boltzmann machine indicates the stability of the state of the machine. In a binary Boltzmann machine, the neurons store a {−1, 1} string, which is referred to as a state. Each state is associated with a value of the energy function. If the Boltzmann machine is initialized with a random state, the tendency of the Boltzmann machine is to move from the current state to a new state that has a lower energy.

A Boltzmann machine is considered already trained or already designed. The weights and biases are fixed. This machine “remembers” some “good” stable states. The “bad” states are unstable, and the Boltzmann machine tries to avoid them. The machine will transition to a stable state every time the current state somehow becomes unstable. In this sense, the energy function for a Boltzmann machine is an objective function, which pushes the neuron state to the closest local minimum. This point will be illustrated by examples later in this paper.

**Figure 2:**
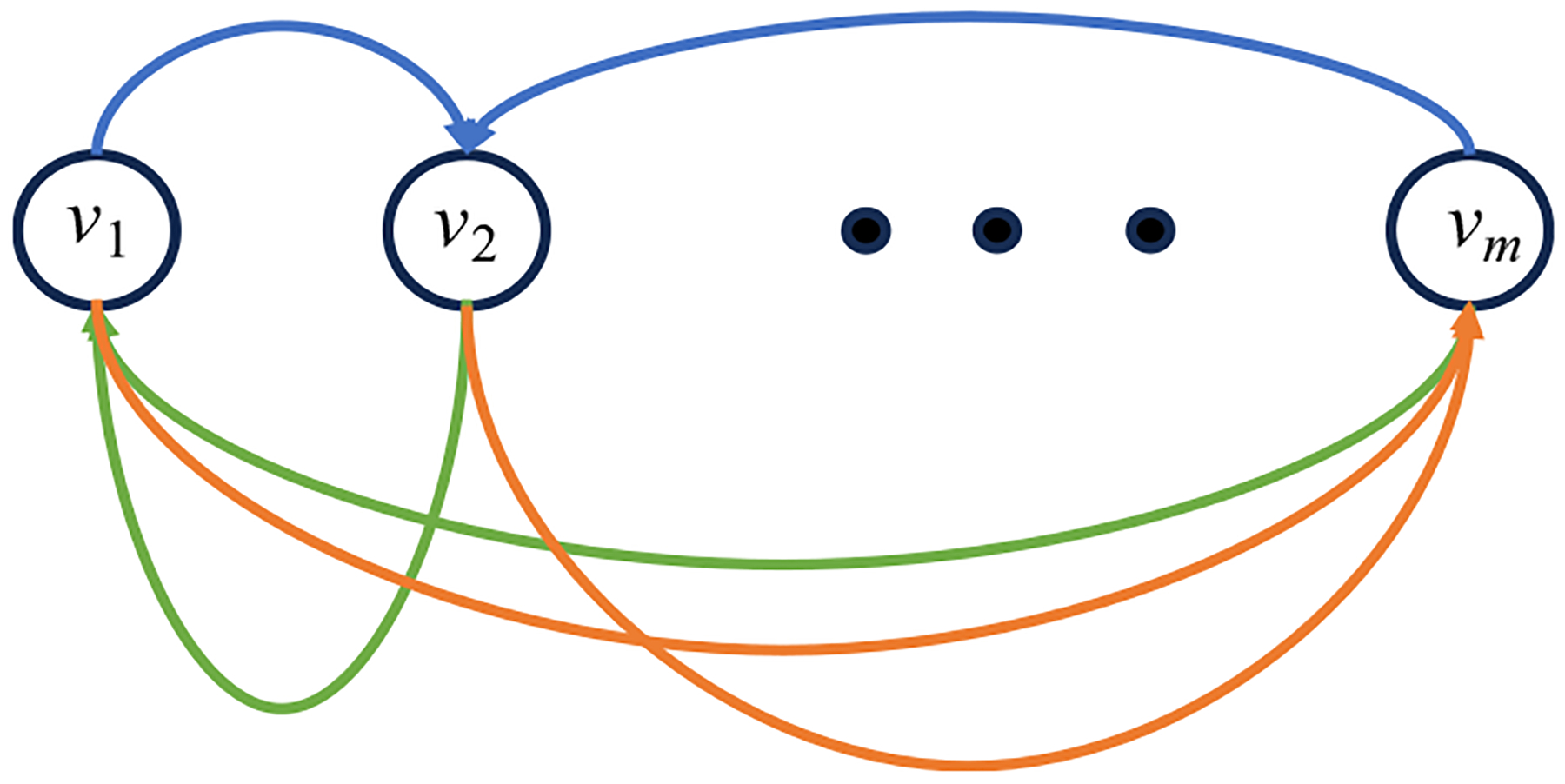
A Hopfield network consists of only one layer

Let us consider a special case of the RBM, in which the visible layer and the hidden layer are the same, that is, h=v. This special case is referred to as the Hopfield network [24] or the Ising model [20] as shown in Figure 2, where the visible layer and the hidden layer merge into one layer. The Hopfield network is fully interconnected, meaning that each neuron (i.e., unit) is connected to every other neuron (unit). These connections have values of $w_{ij}$ from unit i to unit j. The weights typically have the following restrictions:

(1) $$w_{ii}=0,$$

(2) $$w_{ij}=w_{ji}.$$

In other words, no unit has a connection with itself, and connections are symmetric. Each neuron has its own bias and an activation function. We can use a symmetric matrix W to represent the weights in (1) and (2). The diagonal elements of W are zeros.

The network is continuously updating itself. The value of each unit $v_{j}$ is determined by the current values of other units. Unlike a usual perceptron, in which the output is evaluated as an activation function of the inner product of the neuron values from the previous layer and the layer weights plus a bias, the next value of a particular neuron is updated by a not-so-easy algorithm that will be discussed in the next section of this paper. The aim of this algorithm is to select the neuron value such that the energy function of the network is reduced.

For each set of weights $w_{ij}$ and biases $b_{i}$, an energy function E is defined as

(3) $$E=-\frac{1}{2}\underset{i,j}{\sum }w_{ij}v_{i}v_{j}-\underset{i}{\sum }b_{i}v_{i}$$

When the weights and biases are given and fixed, the neuron values will get updated by an algorithm, and the network will eventually converge to a local minimum of the energy function E. In a Hopfield network, we do not see the usual activation functions; instead, we use an algorithm to generate a new state of the neurons.

When we say that the network will converge to a lower-energy state, we do not mean to update the weights and biases. The network reaches a lower-energy state by updating the values of the neurons. The weights and biases cannot be changed. The determination (i.e., training) of the weights and biases is not easy and is beyond the scope of this paper [25].

## 2. Methods

A Hopfield network is fully determined by the weights and biases. The weights and biases then uniquely determine an energy function E as given by (6). The local minima of the energy function E are the stable “solutions” that are stored in the Hopfield network [26, 27]. After Hopfield network neurons are initialized by a random binary string, the network will gradually converge to its closest local “solution” by an algorithm. We are now deriving such an algorithm as follows. This algorithm is going to be nondeterministic.

Traditionally, finding a local minimum in a Hopfield network is first to represent its energy function E as a joint probability density function of a certain stochastic distribution, which is called Boltzmann probability distribution as

(4) $$P\left(\text{state}\right)=\frac{1}{Z}e^{-\frac{E}{T}},$$

where T is the “temperature” and Z is a normalization constant so that the sum of *P*(*state*) is 1 for all possible states. The term “temperature” is a parameter that is used in some optimization algorithms related to the Boltzmann distribution and artificial annealing. This term is borrowed from thermal dynamics. A higher “temperature” indicates a parameter that encourages randomness. The function defined in (4) is monotonically decreasing. It is important to notice that a large E value corresponds to a small P value and a small E value corresponds to a large P value. In other words, minimizing E is equivalent to maximizing P. A state is a possible binary string of $V=\left[v_{1},v_{2},\ldots ,v_{m}\right]$. If E is a non-negative real number, then $P\left(\text{state}\right)$ is in (0, 1] and

(5) $$\underset{\text{state}}{\sum }P\left(\text{state}\;V\right)=1.$$

It is thus justified to treat P as a probability density function of a certain stochastic distribution. Every state is assigned a P value, which is a probability. Minimizing the energy function E is equivalent to finding a state that maximizes the probability density function P locally.

Let $v_{k}^{+}$ denote the state which is equal to V in all positions except at position k (i.e., $v_{i}$ with i≠k) and equal to +1 at position k (i.e., $v_{k}=+1$):

(6) $$v_{k}^{+}=\left(v_{1},\ldots ,v_{k-1},1,v_{k+1},\ldots ,v_{m}\right).$$

Similarly, $v_{k}^{-}$ is defined as

(7) $$v_{k}^{-}=\left(v_{1},\ldots ,v_{k-1},-1,v_{k+1},\ldots ,v_{m}\right).$$

We now define the ratio p of two probabilities as,

(8) $$p=\frac{P\left(v_{k}^{+}\right)}{P\left(v_{k}^{-}\right)}=\frac{e^{-E\left(v_{k}^{+}\right)}}{e^{-E\left(v_{k}^{-}\right)}}=e^{-E\left(v_{k}^{+}\right)+E\left(v_{k}^{-}\right)}.$$

Here, the uppercase P is a probability, and the lowercase p is the ratio of two probabilities. Let us consider the following conditional probability of neuron $v_{k}$ being +1 given the current state of other neurons:

(9) $$P\left(v_{k}=1|v_{i\neq k}\right)\;=\frac{P\left(v_{k}^{+}\right)}{P\left(v_{i\neq k}\right)}\;=\frac{P\left(v_{k}^{+}\right)}{P\left(v_{k}^{-}\right)+P\left(v_{k}^{+}\right)}\;=\frac{P\left(v_{k}^{+}\right)/P\left(v_{k}^{-}\right)}{1+P\left(v_{k}^{+}\right)/P\left(v_{k}^{-}\right)}\;=\frac{p}{1+p}\;=\frac{e^{-E\left(v_{k}^{+}\right)+E\left(v_{k}^{-}\right)}}{1+e^{-E\left(v_{k}^{+}\right)+E\left(v_{k}^{-}\right)}}\;=\frac{e^{-E\left(v_{k}^{+}\right)+E\left(v_{k}^{-}\right)}}{1+e^{-E\left(v_{k}^{+}\right)+E\left(v_{k}^{-}\right)}}.$$

If we introduce the logistic sigmoid function $\varphi \left(z\right)$ as

(10) $$\varphi \left(z\right)=\frac{1}{1+e^{-z}},$$

then

(11) $$P\left(\left.v_{k}=1\right|\text{other}\;v_{i\neq k}\right)=\varphi \left(E\left(v_{k}^{-}\right)-E\left(v_{k}^{+}\right)\right).$$

Consequently, the conditional probability of neuron $v_{k}$ being −1 is given as

(12) $$P\left(v_{k}=-1|\text{other}\;v_{i\neq k}\right)=1-\varphi \left(E\left(v_{k}^{-}\right)-E\left(v_{k}^{+}\right)\right).$$

**Figure 3:**
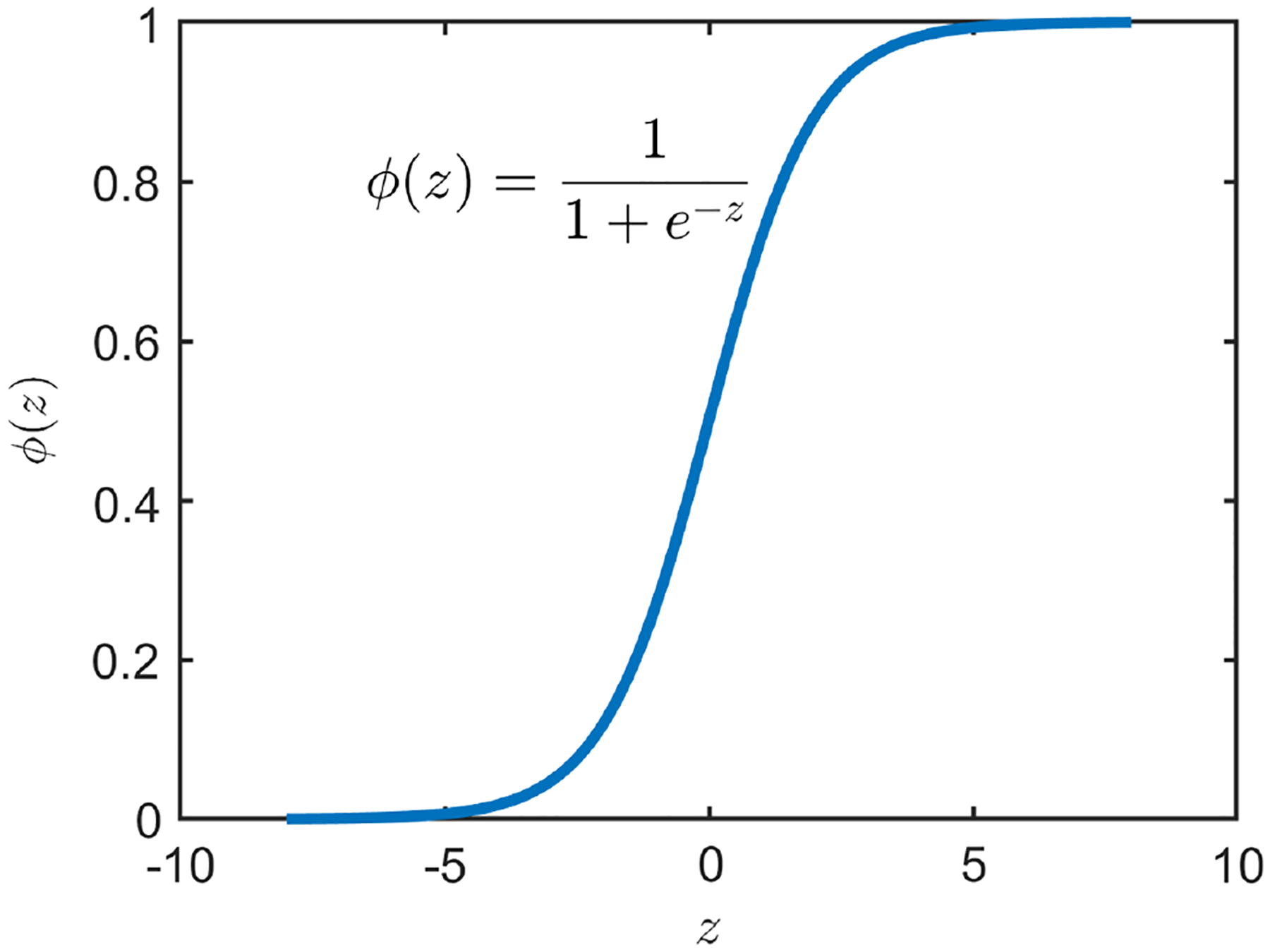
The logistic sigmoid function

Clearly, according to (10), the values of the logistic sigmoid function are between 0 and 1. A plot of the logistic sigmoid function is shown in Figure 3. It is reasonable to treat $P\left(v_{k}=1|v_{i\neq k}\right)$ as a probability.

It is straightforward to observe that if $P\left(v_{k}=1|\text{other}\;v_{i\neq k}\right)>0.5$, then $P\left(v_{k}=1|\text{other}\;v_{i\neq k}\right)<0.5$. In this case, the network has a higher tendency to choose $v_{k}=1$ than to choose $v_{k}=-1$. According to Figure 3 and (10), $P\left(\left.v_{k}=1\right|\text{other}\;v_{i\neq k}\right)>0.5$ implies that $E\left(v_{k}^{+}\right)<E\left(v_{k}^{-}\right)$. Therefore, the choice of $v_{k}=1$ reduces the energy E.

The nondeterministic algorithm can be explained with a small example as follows. We are trying to update the value of the k th neuron $v_{k}$. We first calculate $P\left(\left.v_{k}=1\right|\text{other}\;v_{i\neq k}\right)$ according to (11). Let us assume that this value happens to be 0.7. We then run a random number generator that generates a random number uniformly distributed on [0, 1]. If this random number is < 0.7, we set $v_{k}=1$; otherwise, we set $v_{k}=-1$. The nondeterministic nature of this algorithm is created by the [0, 1] uniform number generator. It allows a probability of 0.7 to set $v_{k}$ to be 1 and a probability of 0.3 to set $v_{k}$ to be −1.

In fact, we do not have to convert the energy function E into a Boltzmann distribution (4). Then we can stay away from any random variables. The updating algorithm will become deterministic as explained as follows.

We now explain the deterministic algorithm using the same small example as above. We are still trying to update the value of the k th neuron $v_{k}$. We first calculate the energy values $E\left(v_{k}^{+}\right)$ and $E\left(v_{k}^{-}\right)$ according to (3), where the exponential function is never used and the energy is never converted into the Boltzmann distribution. If $E\left(v_{k}^{+}\right)<E\left(v_{k}^{-}\right)$, we set $v_{k}=1$; otherwise, we set $v_{k}=-1$.

As a matter of fact, there is an easier way. It can be shown that the energy difference can be easily calculated as [27]

(13) $$E\left(v_{k}^{-}\right)-E\left(v_{k}^{+}\right)=2\left(W_{k}V+B\right)$$

where, using an N-neuron Hopfield network example,

(14) $$V=\left[\begin{array}{c} v_{1}\\ v_{2}\\ \vdots \\ v_{N} \end{array}\right]$$

(15) $$B=\left[\begin{array}{c} b_{1}\\ b_{2}\\ \vdots \\ b_{N} \end{array}\right]$$

(16) $$W=\left[\begin{array}{cccc} 0 & w_{12} & \cdots & w_{1N}\\ w_{12} & 0 & \cdots & w_{2N}\\ \vdots & \vdots & \ddots & \vdots \\ w_{1N} & w_{2N} & \cdots & 0 \end{array}\right]$$

and $W_{k}$ is the k th row of W. The deterministic algorithm can be readily written as

(17) $$v_{k}=\sigma \left(\underset{i}{\sum }w_{ik}v_{i}+b_{j}\right)$$

where σ is an activation sign function:

(18) $$\sigma \left(x\right)=\left\{\begin{array}{ccc} 1 & \text{if} & x>0\\ -1 & \text{if} & x\leq 0. \end{array}\right.$$

It is interesting to notice that the deterministic algorithm (17) happens to be the usual expression for a perceptron, which involves a dot product and bias term and a nonlinear activation function.

The experiment details are given in Tables 1 and 3, which show the logical relationship between the inputs and the output. The inverse solutions (i.e., the inputs) for a given output in general is not unique. Mathematically speaking, the inverse function really does not exist because the function value is not unique. If the “inverse function” has three values, these three values should have an equal chance to reach. The multiple solutions are the local minima of an objective function which is defined by the matrices W and B. The proposed deterministic optimization algorithm is iteratively updating the variables ($v_{1}$ and $v_{2}$, in our examples) according to (17).

Instead of using (17), an alternative way to update a variable $v_{k}$ is the direct usage of the objective function E as follows:

(19) $$v_{k}=\left\{\begin{array}{ccc} 1 & \text{if} & E\left(v_{k}=1\right)<E\left(v_{k}=-1\right)\\ -1 & \text{if} & E\left(v_{k}=1\right)>E\left(v_{k}=-1\right) \end{array}\right.$$

where the objective function E is evaluated using the current variable values other than $v_{k}$.

In the deterministic algorithm, the concept of the probability distribution is never used. The entire Hopfield network updating procedure is deterministic. Some numeric examples are presented to compare these two methods in the next section.

## 3. Results

This section uses two examples to illustrate the performance differences between a nondeterministic algorithm and a deterministic algorithm in Hopfield network applications. In these two examples, the Hopfield networks are assumed to be already designed. In other words, the weights W and biases B are given. The goal is to find the closest local minimum of the energy function.

In the first example, we use a Hopfield network to remember the stable OR gate states using {−1, 1} as variables. The logic OR gate relations are defined in Table 1.

The four stable states listed in Table 1 are the four lower-energy states with negative energy values in an energy function E defined in (3), where the weights and biases are the elements of W and B given as

(20) $$W=\left[\begin{array}{ccc} 0 & -1 & 2\\ -1 & 0 & 2\\ 2 & 2 & 0 \end{array}\right]$$

and

(21) $$B=\left[\begin{array}{c} -1\\ -1\\ 2 \end{array}\right]$$

The stable states are (−1, −1, −1), (−1, 1, 1), (1, −1, 1), and (1, 1, 1).

We now verify that the energy function defined by (20) and (21) indeed has the local minima for the desired stable OR gate states and has unstable larger energy values for the states that do not satisfy the OR gate relationship. There are eight possible states. Four of the states are stable, and the other four states are unstable as listed in Table 2, as calculated by using (3).

**Figure 4:**
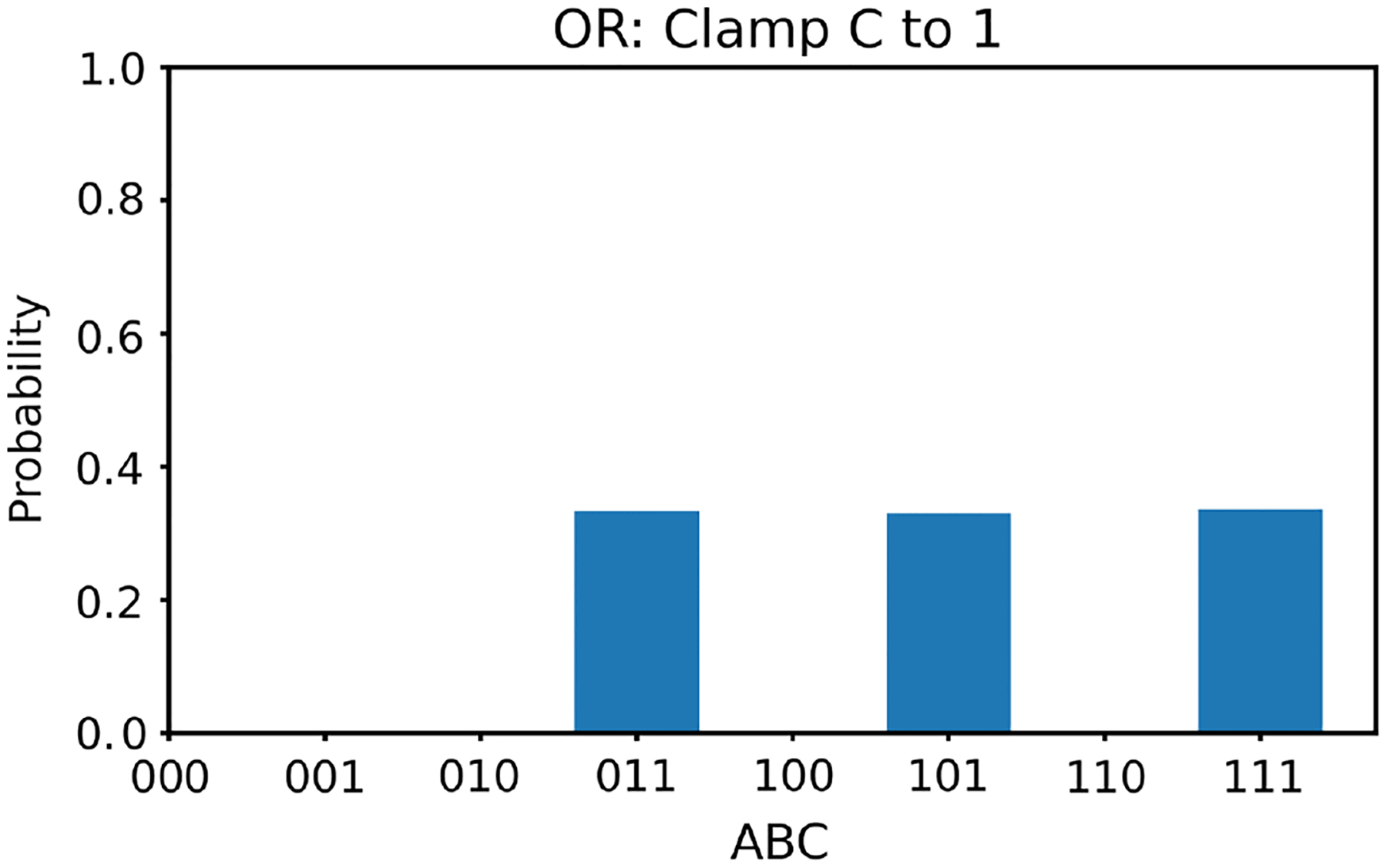
The histogram of the Hopfield network converged states for the OR gate model with $v_{3}$ clamped to +1 using the deterministic algorithm

**Figure 5:**
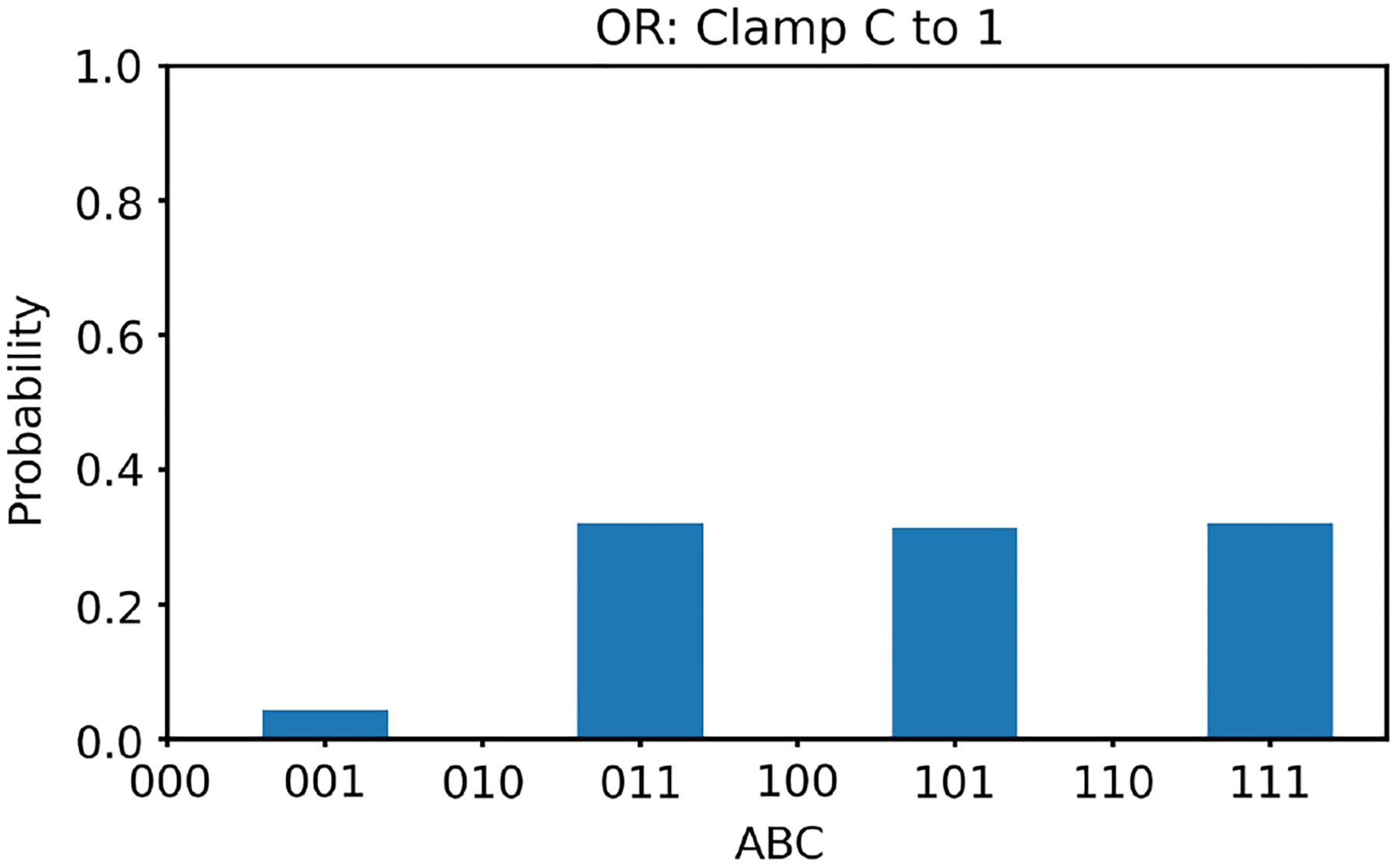
The histogram of the Hopfield network converged states for the OR gate model with $v_{3}$ clamped to +1 using the nondeterministic algorithm

We first clamp the value of $v_{3}$ to +1. In this case, according to Table 1, there are three solutions for $\left(v_{1},v_{2}\right)$; they are (−1, 1), (1, −1), and (1, 1). With $v_{3}$ clamped as +1, we generate 100,000 random initial values of $\left(v_{1},v_{2}\right)$ and run the two energy minimization algorithms (one deterministic and one nondeterministic), respectively. For each algorithm, we obtain 100,000 converged states. The converged states are summarized in a histogram. The histogram results are shown in Figures 4 and 5, respectively. The ideal result should show the equal likelihood of the solutions of (−1, 1),(1, −1), and (1, 1). There should be very little occurrence elsewhere. It can be observed that the deterministic algorithm gives much better results than the nondeterministic algorithm. In the figures, “−1” is labeled as “0” along the horizontal axes, and $v_{1}v_{2}v_{3}$ is labeled as ABC with $A=v_{1},\;B=v_{2}$, and $C=v_{3}$.

**Figure 6:**
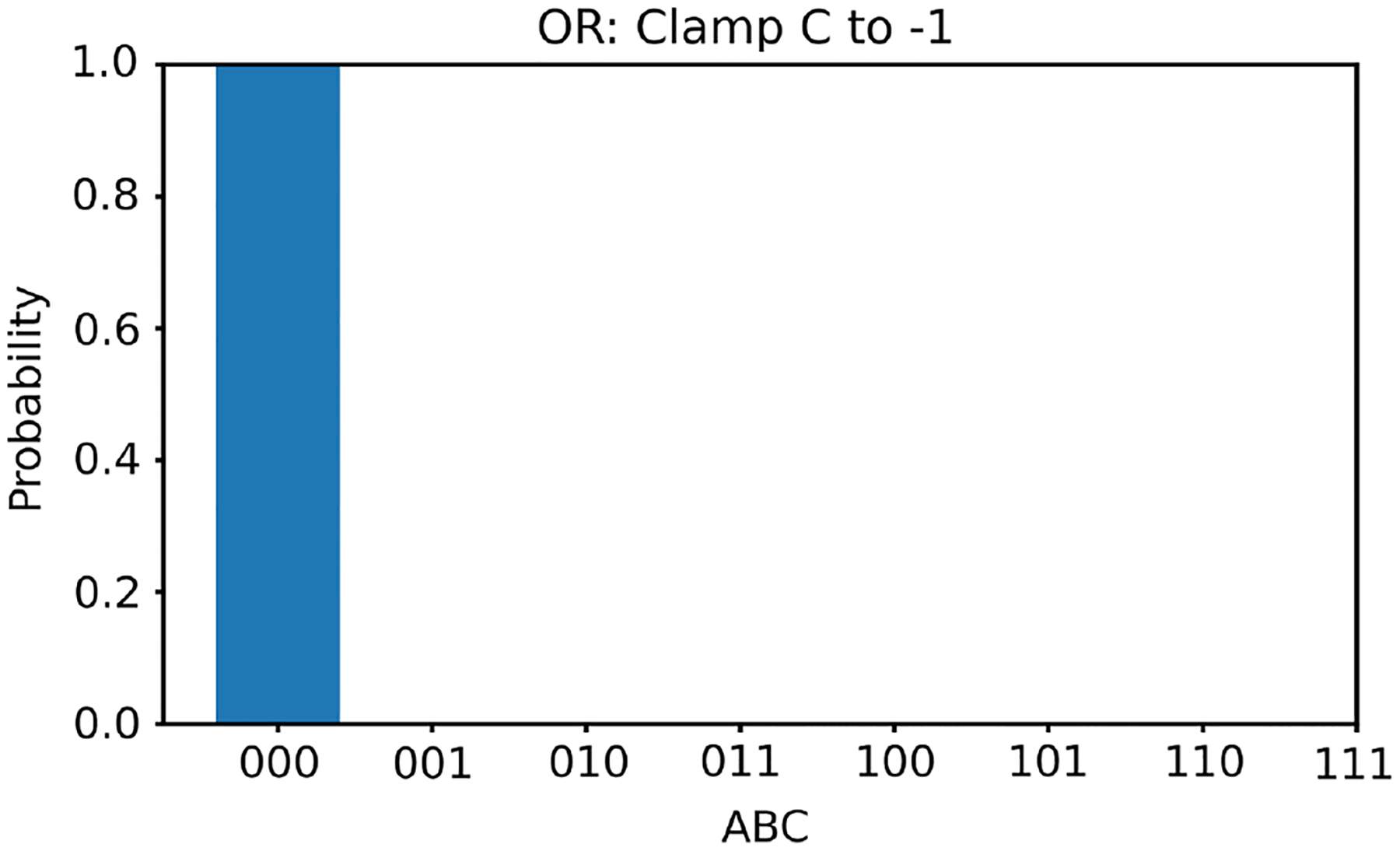
The histogram of the Hopfield network converged states for the OR gate model with $v_{3}$ clamped to −1 using the deterministic algorithm

**Figure 7:**
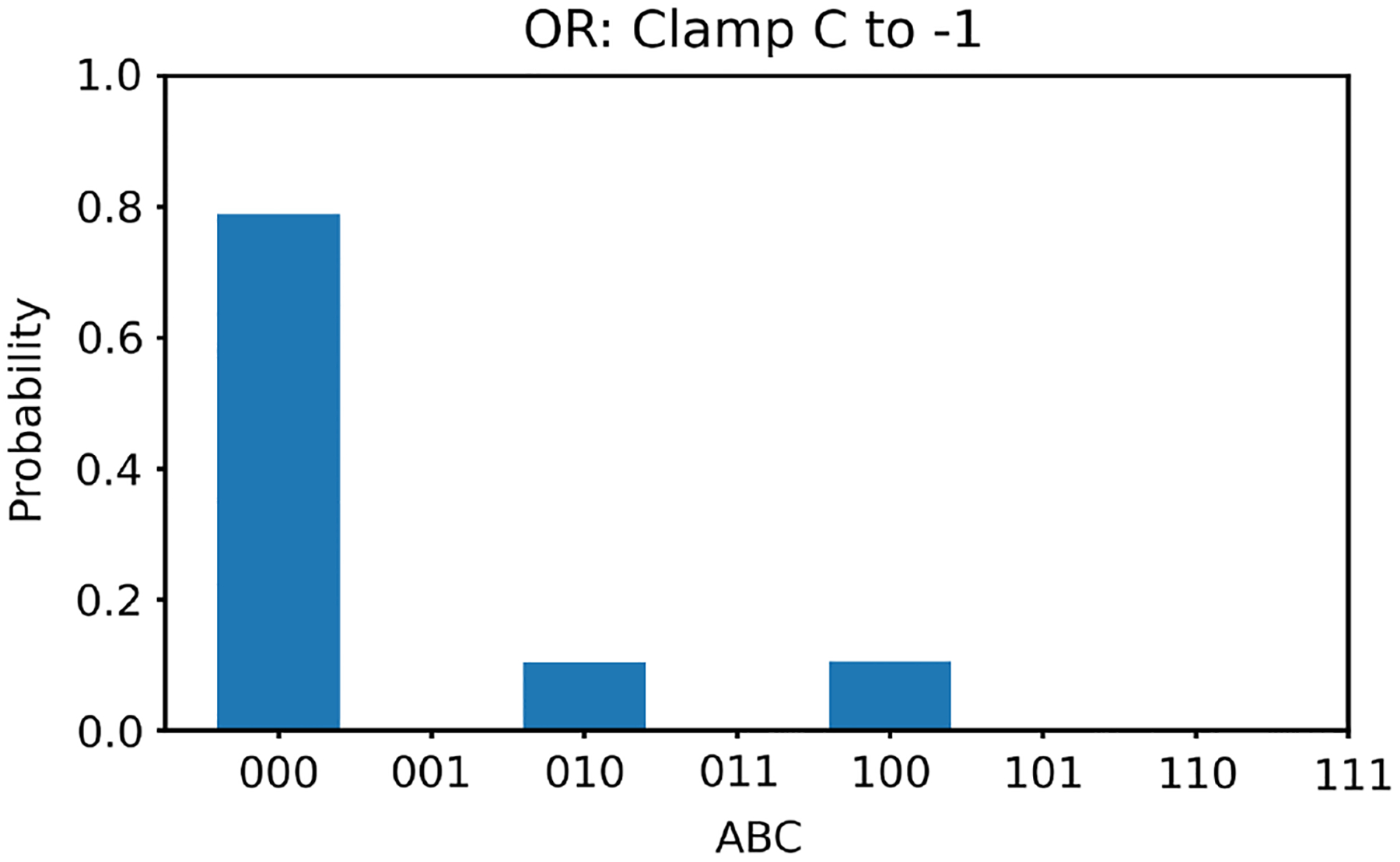
The histogram of the Hopfield network converged states for the OR gate model with $v_{3}$ clamped to −1 using the nondeterministic algorithm

Next, we clamp the value of $v_{3}$ to −1. In this case, according to Table 1, there is only one solution for $\left(v_{1},v_{2}\right)$, that is, (−1, −1). With $v_{3}$ clamped to −1, we generate 100,000 random initial values of $\left(v_{1},v_{2}\right)$ and run the two energy minimization algorithms (one deterministic and one nondeterministic), respectively. The histogram results are shown in Figures 6 and 7, respectively. The ideal result should only show (−1, −1) with nothing elsewhere. Once again, the deterministic algorithm gives much better results than the nondeterministic algorithm.

We can repeat the same studies for the logic AND gate using {−1, 1}. The AND gate logic relationships are listed in Table 3.

The four stable states listed in Table 3 are the four lower-energy states with negative energy values in an energy function E defined in (3) whose W and B are given as

(22) $$W=\left[\begin{array}{ccc} 0 & -1 & 2\\ -1 & 0 & 2\\ 2 & 2 & 0 \end{array}\right]$$

and

(23) $$B=\left[\begin{array}{c} 1\\ 1\\ -2 \end{array}\right].$$

The stable states are (−1, −1, −1), (−1, 1, 1),(1, −1, 1), and (1, 1, 1).

It is interesting to notice that the W matrices in (20) and (22) are the same. However, the B vector for the AND gate is the negative counterpart of the B vector for the OR gate.

We now verify that the energy function defined by (22) and (23) indeed has the local minima for the desired stable AND gate states and has unstable larger energy values for the states that do not satisfy the AND gate relationship. There are eight possible states. Four of the states are stable, and the other four states are unstable as listed in Table 4, as calculated by using (3).

**Figure 8:**
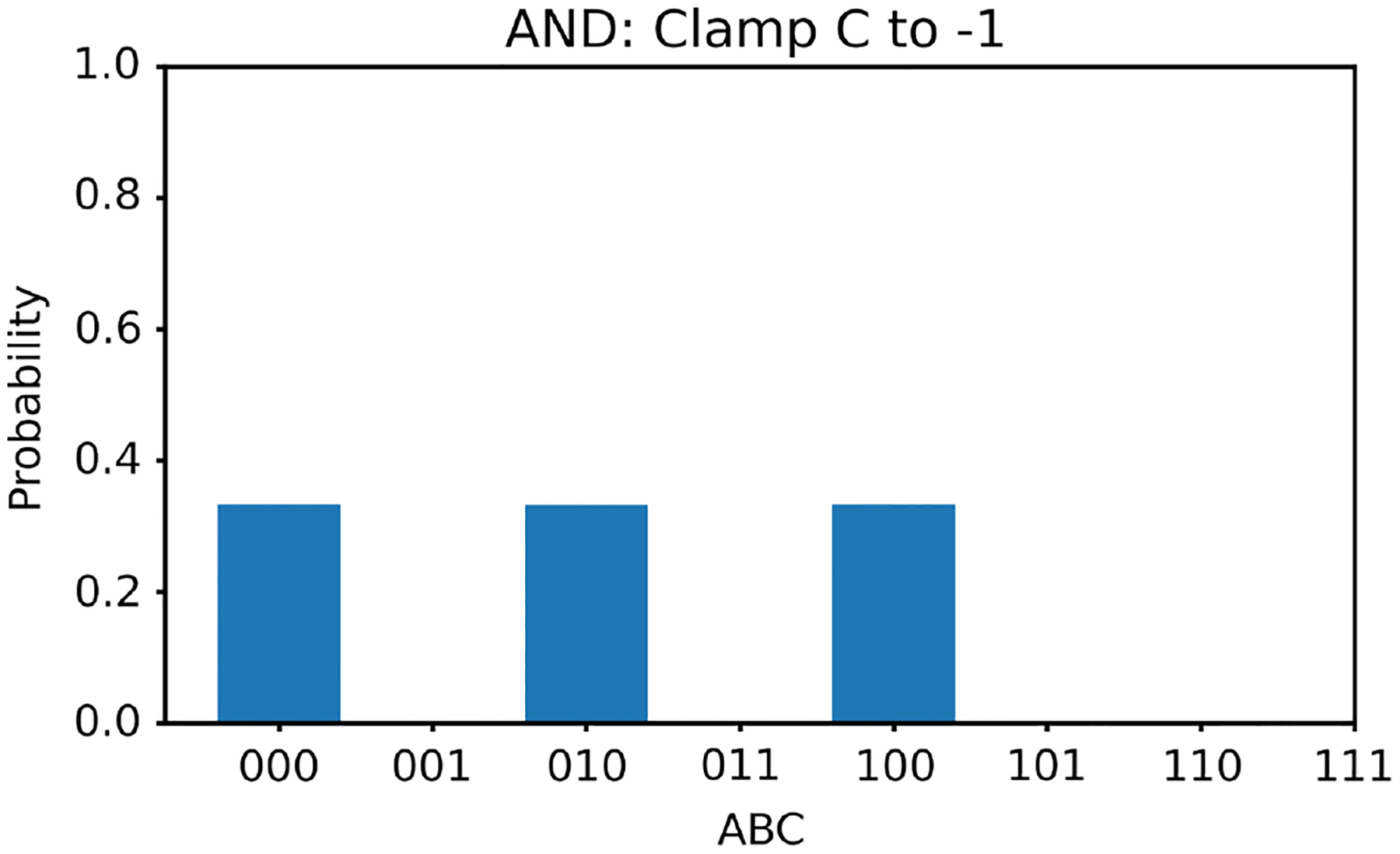
The histogram of the Hopfield network converged states for the AND gate model with $v_{3}$ clamped to −1 using the deterministic algorithm

**Figure 9:**
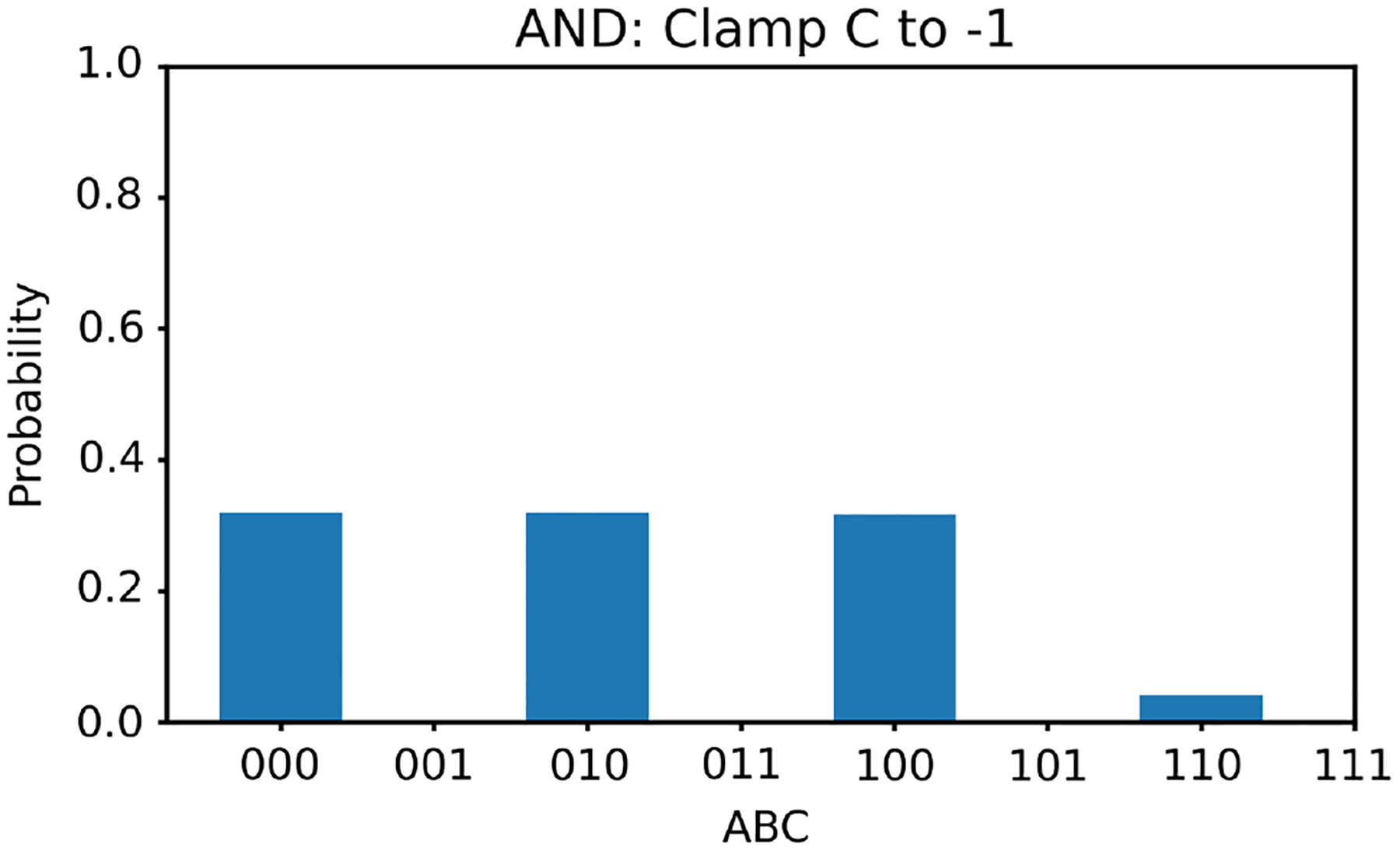
The histogram of the Hopfield network converged states for the AND gate model with $v_{3}$ clamped to −1 using the nondeterministic algorithm

Similar to the OR gate study, we first clamp the value of $v_{3}$ to −1. In this case, according to Table 3, there are three solutions for $\left(v_{1},v_{2}\right)$; they are (−1, −1), (1, −1), and (−1, 1). With $v_{3}$ clamped to −1, we generate 100,000 random initial values of $\left(v_{1},v_{2}\right)$ and run the two energy minimization algorithms (one deterministic and one nondeterministic), respectively. The histogram results are shown in Figures 8 and 9, respectively. The ideal result should show the equal likelihood of the solutions of (−1, −1), (1, −1), and (−1, 1). There should be very little occurrence elsewhere. The deterministic algorithm gives much better results than the nondeterministic algorithm.

**Figure 10:**
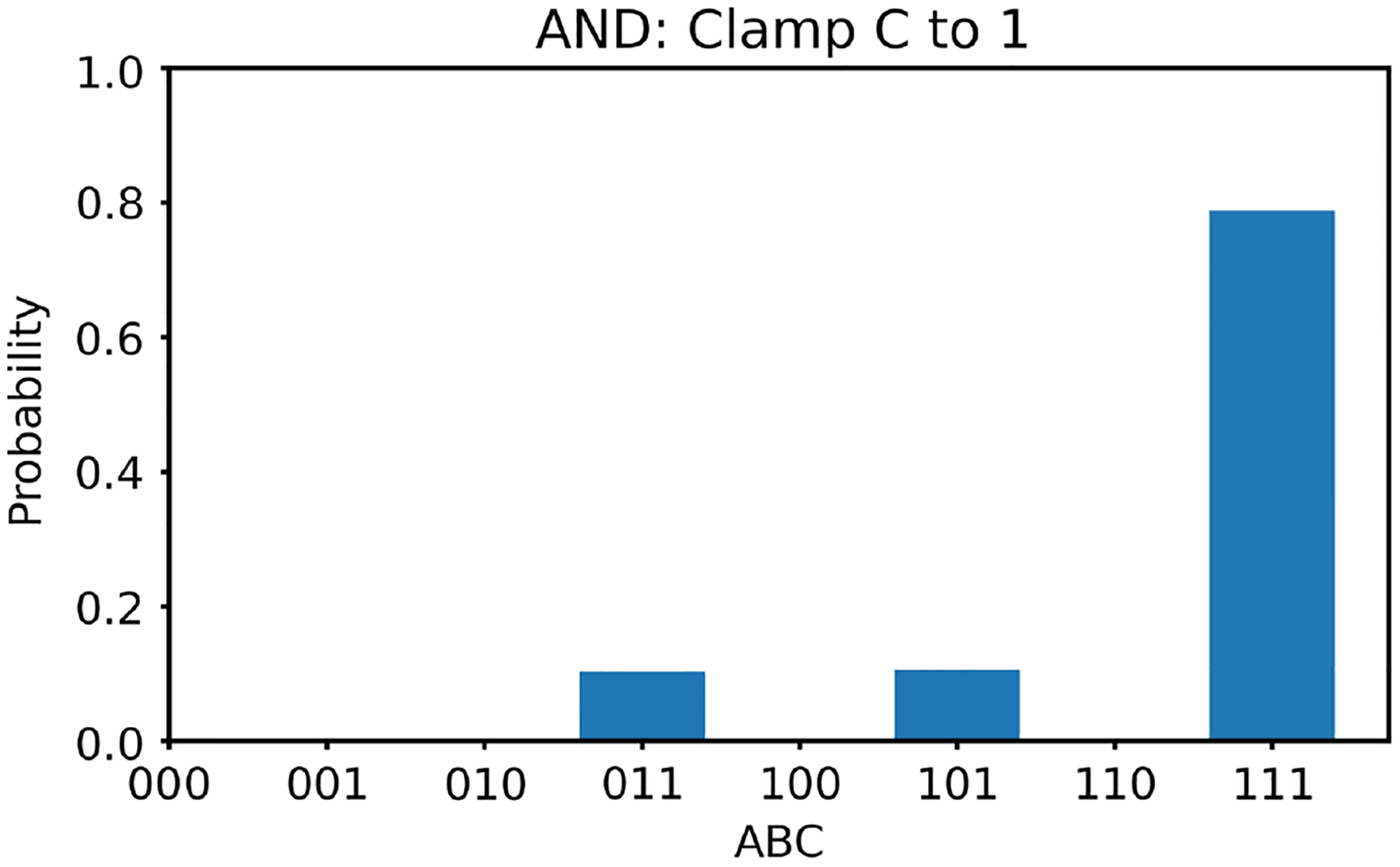
The histogram of the Hopfield network converged states for the AND gate model with $v_{3}$ clamped to +1 using the deterministic algorithm

**Figure 11:**
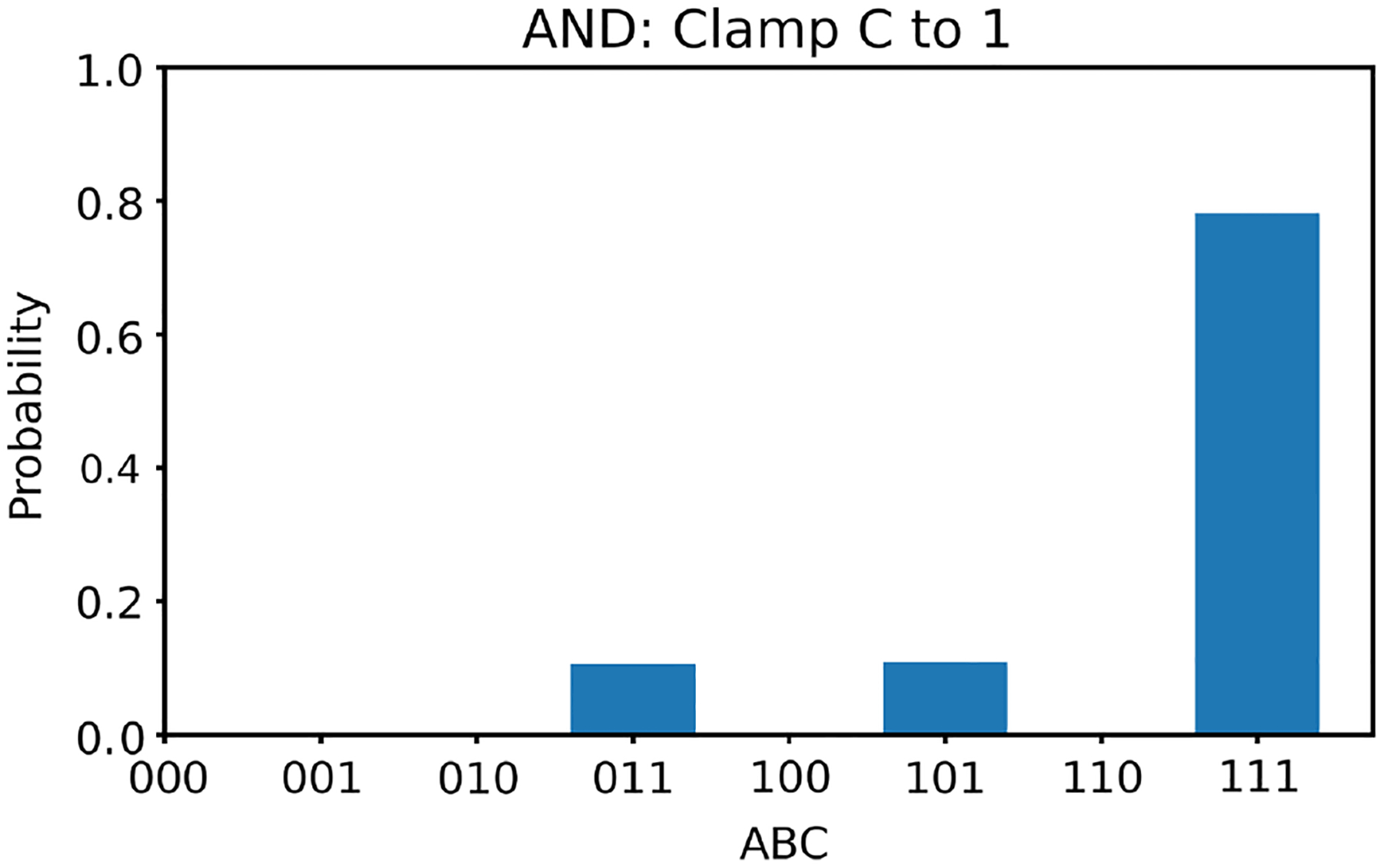
The histogram of the Hopfield network converged states for the AND gate model with $v_{3}$ clamped to +1 using the nondeterministic algorithm

Next, we clamp the value of $v_{3}$ to +1. In this case, according to Table 2, there is only one solution for $\left(v_{1},v_{2}\right)$, that is, (1, 1). With $v_{3}$ clamped as +1, we generate 100,000 random initial values of $\left(v_{1},v_{2}\right)$ and run the two energy minimization algorithms (one deterministic and one nondeterministic), respectively. The histogram results are shown in Figures 10 and 11, respectively. The ideal result should only show (1, 1) with nothing elsewhere. Once again, the deterministic algorithm gives much better results than the nondeterministic algorithm.

## 4. Discussion

This paper focuses on the theoretical problem of local optimization. In the two examples, we have two problems. One problem is to find the inverse function of an AND gate logic. The other problem is to find the inverse function of an OR gate logic. These two inverse functions are used to form two objective functions, which have multiple local minima. These two objective functions are distinct, and their local minima are different. Our computer simulations demonstrate that for an objective function with multiple local minima, the proposed method is not biased if the initial condition is uniformly sampled.

An NP-hard problem is to search for the global solution. Our method is a greedy method, which looks for the nearest local minimum. In this paper, both deterministic and nondeterministic methods are greedy methods. To search for the local minimum, the iterative algorithms (similar to the gradient descent algorithms) take many steps to reach the local minimum. Since the nondeterministic algorithm allows a percentage of the total number of steps to move in the uphill direction, it will take more steps to converge. An error in the context of the current paper is referred to the situation where the algorithm converges to a local minimum that is not the nearest one.

The implementation of the proposed deterministic algorithm is fairly straightforward for a binary problem, in which the variables can only take two values. On the other hand, in an optimization algorithm with continuous variables, the update step size is tricky to select. If the step size is too large, the algorithm may diverge. If the step size is too small, the algorithm may take too long to converge. For a binary optimization algorithm, a variable may stay at its current value or change to the other value. The most difficult part of using an RBM to solve a practical problem is to set up the objective function.

For deterministic optimization, the objective function is the same as the energy function. No conversion is needed. On the other hand, for the nondeterministic optimization, the energy function must be mapped to the range of [0, 1] to treat it as a probability distribution.

For any discrete problem, for example, integer programming and binary programming, a local minimum is perfectly reached. However, to find the global minimum requires a brute-force search and is well-known to be NP-hard.

The deterministic optimization finds the nearest local minimum from the initial condition. In order to find the global optimum, one must start with a large number of different initial conditions and compare their results.

For binary Boltzmann machines, the variables can only take two values. Therefore, the activation function can only take two values. Thus, the step function is a natural choice for the activation function.

## 5. Conclusions

An RBM is a fully connected neural network consisting of two layers: the visible layer and the hidden layer, respectively. A common RBM is binary valued and associated with an energy function, which is a quadratic form. When these two layers are the same, the RBM is reduced to a Hopfield network. In this paper, we consider a binary Hopfield network, where the neurons take the values of {−1, 1}. However, the weights and biases can take any real numbers.

In a Hopfield network, the weights and biases are designed by a problem at hand. The Hopfield network is not trained. For example, the weights can be calculated by the Hebbian learning rule [28]. Once the Hopfield network is designed, it can remember some good stable solutions. Algorithms are available to update the state of the Hopfield network so that a random state can converge to a local minimum of the energy function.

The traditional method is to represent this energy function as a joint probability distribution by using an exponential function conversion. Minimizing the energy function is equivalent to iteratively increasing the conditional probability for each unit (i.e., neuron).

For a binary Hopfield network, at each iteration step, a unit has a probability, say q, to change to 1. The state of a unit is a random variable, and its update procedure is implemented randomly according to the conditional probability q. Therefore, the iterative algorithm is nondeterministic.

This paper uses a different method to update a binary Hopfield network’s neuron value. We do not convert the energy function into a joint probability distribution. We use the original definition of the network energy. The optimization of the energy function is now a deterministic problem. The optimization procedure is still iterative but no longer nondeterministic. The proposed deterministic procedure has advantages over the nondeterministic procedure: faster convergence and smaller errors. Best of all, the deterministic update algorithm is in the standard form of a typical perceptron, which consists of a dot product, a bias, and a nonlinear activation function.

The main goal of a Hopfield network is to search for local minima of an energy function. Finding the local minima of an energy function is a mathematical problem. In our manuscript, the proposed deterministic method is applied to four problems: an inversion of the AND gate function with the output clamped to 1, an inversion of the AND gate function with the output clamped to −1, an inversion of the OR gate function with the output clamped to 1, and an inversion of the OR gate function with the output clamped to −1. The proposed deterministic method is compared with the more popular nondeterministic method using these four applications in the form of histograms in the “Results” section. The deterministic method can be applied to many other optimization problems, especially the NP-hard problems, such as number partition, graph partitioning, cliques, satisfiability, minimal maximal matching, graph coloring, tree problems, knapsack with integer weights, and binary integer linear programming, to name a few [25]. This paper presents some computer simulations to compare these two methods. The deterministic algorithm demonstrates more advantages than the nondeterministic algorithm.

## Figures and Tables

**Table 1:** The logic OR gate in {−1, 1}

$v_{1}$	$v_{2}$	$v_{3}$
−1	−1	−1
−1	1	1
1	−1	1
1	1	1

**Table 2:** The energy values for all possible states by using the logic OR gate relationship in {−1, 1}

	$v_{1}$	$v_{2}$	$v_{3}$	E	
An OR gate state	−1	−1	−1	−3	Stable
An OR gate state	−1	1	1	−3	Stable
An OR gate state	1	−1	1	−3	Stable
An OR gate state	1	1	1	−3	Stable
A non-OR gate state	−1	−1	1	1	Unstable
A non-OR gate state	−1	1	−1	1	Unstable
A non-OR gate state	1	−1		1	Unstable
A non-OR gate state	1	1		9	Unstable

**Table 3:** The logic AND gate in {−1, 1}

$v_{1}$	$v_{2}$	$v_{3}$
−1	−1	−1
−1	1	−1
1	−1	−1
1	1	1

**Table 4:** The energy values for all possible states by using the logic AND gate relationship in {−1, 1}

	$v_{1}$	$v_{2}$	$v_{3}$	E	
An AND gate state	−1	−1	−1	−3	Stable
An AND gate state	−1	1	−1	−3	Stable
An AND gate state	1	−1	−1	−3	Stable
An AND gate state	1	1	1	−3	Stable
A non-AND gate state	−1	−1	1	9	Unstable
A non-AND gate state	−1	1	1	1	Unstable
A non-AND gate state	1	−1	1	1	Unstable
A non-AND gate state	1	1	−1	1	Unstable

## Data Availability

Data sharing is not applicable to this article as no new data were created or analyzed in this study.
